# Using fecal immunochemical tubes for the analysis of the gut microbiome has the potential to improve colorectal cancer screening

**DOI:** 10.1038/s41598-021-99046-w

**Published:** 2021-10-01

**Authors:** Kertu Liis Krigul, Oliver Aasmets, Kreete Lüll, Tõnis Org, Elin Org

**Affiliations:** 1 Institute of Genomics, Estonian Genome Centre, University of Tartu, 51010 Tartu, Estonia; 2 Department of Biotechnology, Institute of Molecular and Cell Biology, University of Tartu, Tartu, Estonia

**Keywords:** Bacterial genetics, Cancer prevention, Bacterial genetics

## Abstract

Colorectal cancer (CRC) is a challenging public health problem which successful treatment depends on the stage at diagnosis. Recently, CRC-specific microbiome signatures have been proposed as a marker for CRC detection. Since many countries have initiated CRC screening programs, it would be useful to analyze the microbiome in the samples collected in fecal immunochemical test (FIT) tubes for fecal occult blood testing. Therefore, we investigated the impact of FIT tubes and stabilization buffer on the microbial community structure evaluated in stool samples from 30 volunteers and compared the detected communities to those of fresh-frozen samples, highlighting previously published cancer-specific communities. Altogether, 214 samples were analyzed by 16S rRNA gene sequencing, including positive and negative controls. Our results indicated that the variation between individuals was greater than the differences introduced by the collection strategy. The vast majority of the genera were stable for up to 7 days. None of the changes observed between fresh-frozen samples and FIT tube specimens were related to previously identified CRC-specific bacteria. Overall, we show that FIT tubes can be used for profiling the microbiota in CRC screening programs. This circumvents the need to collect additional samples and can possibly improve the sensitivity of CRC detection.

## Introduction

Colorectal cancer (CRC) affects millions of people worldwide each year and is one of the leading causes of cancer death. Epidemiological data suggest that the incidence of CRC is expected to increase 60% by 2030 due to population aging and the growing popularity of Western diets and lifestyles^1^. Moreover, recent data indicate that the incidence of CRC is increasing, especially among younger adults^2^. Therefore, CRC has become an important and challenging global public health problem, in which the detection of cancer in early stages is of high importance. The development of CRC is a stepwise process; CRC can be prevented by the detection and removal of adenomas and serrated polyps before they become cancerous^3^. Therefore, many countries throughout Europe and the world have started inviting individuals in the age range of 50–74 years to participate in population-based screening programs to increase early CRC detection by analyzing fecal blood via a fecal immunochemical test (FIT), followed by colonoscopy.

Although FIT-based screening reduces both the incidence and mortality of CRC and is cost effective, current screening programs face multiple challenges. The participation rate in FIT-based screening programs varies widely across different countries, ranging from 22.8 to 71.3% in Europe^4^. Furthermore, commonly used FITs have shown low sensitivity for precancerous lesions (sensitivity for sessile-serrated adenomas/polyps is 12.3% and for advanced adenomas is 32.4%)^5^ and early-stage cancer (sensitivity for stage T1 is 40%)^6^. Additionally, FITs may show false-negative results due to smoking or advanced age, both of which are well-known risk factors for CRC^7^, causing some cases to be missed. Moreover, data have shown that over 20% of colorectal adenomas can be missed in colonoscopy, which is considered the gold standard for CRC diagnosis^8,9^. Finally, approximately 30% of FIT-positive (cutoff of ≥ 20 μg of hemoglobin/mg of feces) individuals undergoing colonoscopy might show negative colonoscopy results (i.e., a normal colon without any pathologies)^10^. Taken together, a new screening test can only be successful if the participation rate and colonoscopy quality of the screening program are high. Consequently, new highly specific, inexpensive, and sensitive noninvasive screening tests that improve the detection of precancerous colorectal lesions and CRC without compromising the programs participation rate are urgently needed to further reduce CRC incidence and mortality.

Accumulating evidence indicates that the microbiome substantially contributes to the development of CRC. Cross-sectional multipopulation studies in humans in which the CRC microbiome^11^ and cancer stage-specific microbial signatures^12^ have been detected in the stool samples of CRC patients have shown significant associations between the gut microbiota and CRC. Therefore, it has been suggested that complementing fecal occult blood tests with gut microbiota analysis could improve the detection of CRC relative to the use of the fecal occult blood test alone^13,14^. As both tests use fecal samples, it would be favorable to obtain both fecal occult blood test and microbiome composition results from the same sample.

It has been recently demonstrated that the fecal immunochemical test (FIT) tubes used for fecal occult blood sample collection have the potential to also be used for sample collection for microbiome studies^15,16^. Many different types of FIT tubes are available on the market, which have different compositions and have been shown to perform differently in the analysis of fecal occult blood from CRC patients^17^, indicating the possibility that they might also vary in performance regarding microbiome detection. CRC screening programs in multiple European countries (e.g., Sweden, Finland, Estonia, Czech Republic, and Slovakia) use the QuikRead® iFOB Sampling Set (Aidian, Espoo, Finland); however, these particular FIT tubes have not yet been tested for use in microbiome sample collection and analysis. Using fecal samples not originally intended for microbial profiling may introduce technical challenges due to incompatible materials and varying sample handling and storage conditions. Therefore, the various ways in which sample processing and storage may potentially influence the microbiota need to be investigated.

In the current study, we aimed to explore the potential to determine the gut microbiome composition from samples collected in QuikRead® iFOB Sampling Set FIT tubes. To this end, we analyzed fecal samples from 30 volunteers stored in FIT tubes and compared them with samples stored via two other methods: fresh-frozen samples and samples stored in DNA/RNA Shield stabilizing solution (Zymo Research, Irvine, California). We investigated how storage in FIT tubes or DNA/RNA Shield stabilizing solution affected gut microbiome diversity and composition estimates as well as community structure stability over time relative to the gold standard of immediate freezing.

## Results

### Study design

**Figure 1:**
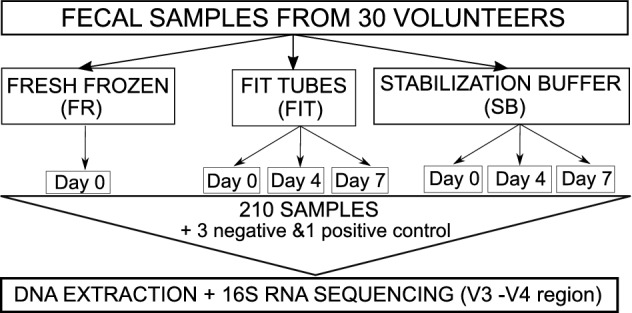
Workflow for sample collection and analysis.

**Figure 2:**
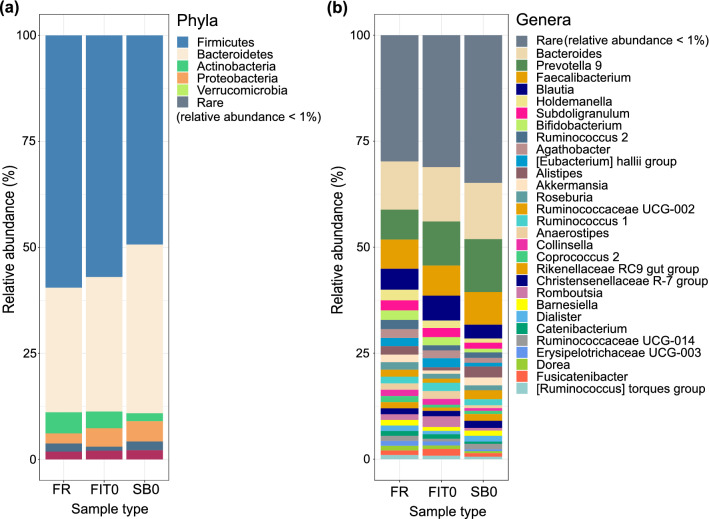
Relative abundance of phyla (**a**) and genera (**b**) under different collection methods on day 0. Taxa with a mean relative abundance of less than 1% were grouped into the rare category. *FR* fresh-frozen; *FIT0* immediately frozen FIT samples; *SB0* immediately frozen stabilization buffer samples.

For each individual in the study, seven stool samples were collected and stored using 3 different methods prior to DNA extraction: 1) fresh, immediately frozen stool samples (FR samples); 2) stool stored in FIT tubes, which was immediately frozen (FIT0) or stored for 4 days (FIT4) or 7 days (FIT7) at room temperature; and 3) stool stored in DNA/RNA shield stabilization buffer, which was immediately frozen (SB0) or stored for 4 days (SB4) or 7 days (SB7) at room temperature. The workflow for the sample collection and storage procedures is shown in Fig. 1. As expected, the highest DNA concentration was obtained from the fresh-frozen samples (mean 374.23 ng/µl), followed by stabilization buffer samples (mean 42.64 ng/µl) and the FIT samples (mean 11.68 ng/µl) (Supplementary Table S1). All samples presented sufficient biomass to obtain enough DNA for 16S sequencing library generation. For each sample, we generated amplicon libraries targeting the bacterial 16S rRNA V3-V4 region, sequenced the libraries, and performed quality filtering and ASV estimation in QIIME (see Methods). Following the QC step, the average number of reads per sample remained relatively stable across all sample types (Supplementary Table S1). The negative controls showed no read counts after the QC step. The MOCK community, which was used as a positive control for sequencing, showed 18 890 reads after the QC step. All genera expected to be present in the positive controls (*Staphylococcus*, *Pseudomonas*, *Enterococcus*, *Escherichia*, *Salmonella*, *Lactobacillus*, *Listeria*, and *Bacillus*) were detected in the analysis (Supplementary Table S2). In total, we detected 11 948 ASVs, 360 genera, 131 families, 64 orders, 32 classes, and 18 phyla. At day 0, all the sample types showed similar taxonomic profiles at both the phylum and genus levels (Fig. 2, Supplementary Table S3). As expected, a Western microbial community structure was observed in all of the sample types, with 90% of bacteria belonging to the phyla *Firmicutes* and *Bacteroides,* followed by the phyla *Proteobacteria*, *Actinobacteria*, and *Verrucomicrobia* (Fig. 2a).

#### Impact of the sample collection strategy on gut microbiome diversity

**Figure 3:**
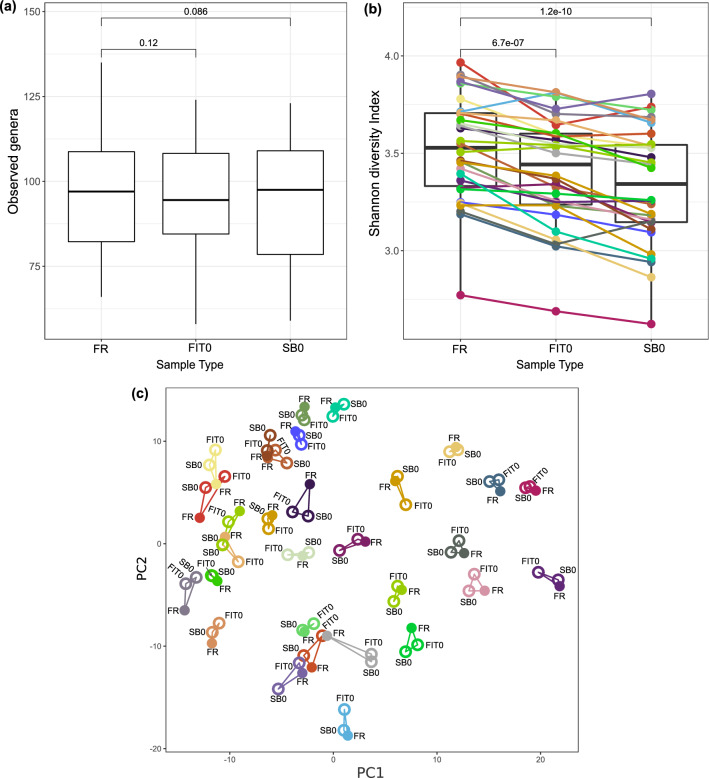
Comparison of microbiome diversity between different sample types. Boxplots represent two different alpha diversity measurements: (**a**) richness or number of taxa observed and (**b**) Shannon diversity index. Median values and interquartile ranges are indicated in the plots. In the richness analysis, paired *t* tests indicated that the differences were not significant (FDR_FR-FIT0_ = 0.12, FDR_FR-SB0_ = 0.086). In the Shannon diversity index plot, samples from the same patient are connected and colored to illustrate the lower trend of alpha diversity for FIT0 and SB0 relative to FR (FDR < 0.01). (**c**) Principal component analysis (PCA) of beta diversity between storage conditions. Samples are colored and linked based on the individual's ID. *FR* fresh-frozen samples; *FIT0* immediately frozen FIT samples; *SB0* immediately frozen stabilization buffer samples.

To evaluate whether the FIT and stabilization buffer samples showed similar diversity to the fresh-frozen samples, we assessed the differences in gut microbiome alpha and beta diversity between the collection methods. Both alpha diversity (richness and Shannon index) and beta diversity metrics were calculated using genus-level transformed data. We detected 98 ± 17.6 genera in the FR samples, 96 ± 16.3 genera in the FIT0 samples, and 95 ± 17.4 in the SB0 samples (Supplementary Table S4). When richness was compared among the sample types, we found that the differences in the observed genera between the fresh samples and the other two sample types were not significant (FDR_FR-FIT0_ = 0.12, FDR_FR-SB0_ = 0.086) (Fig. 3a, Supplementary Table S4). However, the samples stored in FIT tubes and stabilization buffer exhibited lower Shannon index values than the fresh-frozen samples (FR_Shannon_ = 3.5 ± 0.3, FIT0_Shannon_ = 3.4 ± 0.3, SB0_Shannon_ = 3.3 ± 0.3, paired t test FDR < 0.01) (Fig. 3b, Supplementary Table S4). This trend was not only observable between the mean Shannon index values under each storage condition but was also noticeable when each individual’s samples were visualized. When evaluating beta diversity, which represents how much the community changes between sample types, we observed that the samples of the same individual were grouped together regardless of the storage conditions, indicating that the differences between the subjects were greater than the differences between the storage methods (Fig. 3c). When analyzing the significance of variance (PERMANOVA) in samples frozen immediately after collection, we found the sample type to be significant; however, the effect on variance was low (R^2^ = 0.02226, p < 0.001).

#### Differentially abundant genera between sample collection strategies

**Figure 4:**
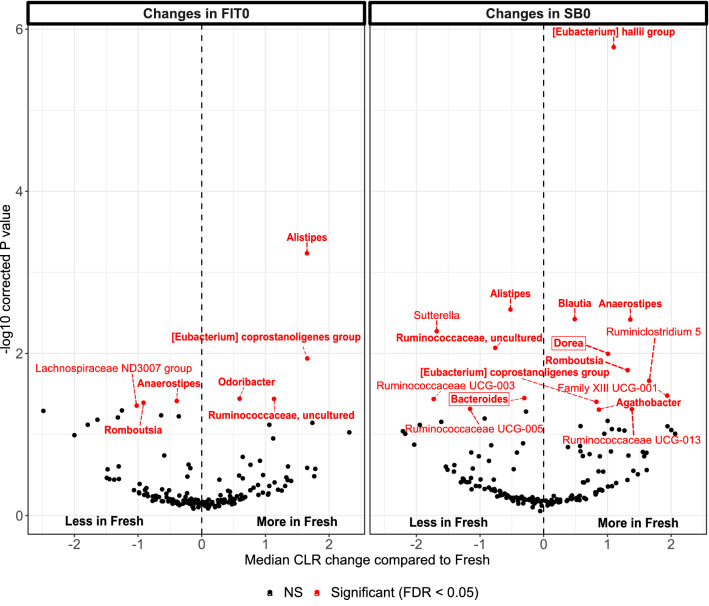
Differentially abundant genera between different sample types. Average CLR changes in FIT0 and SB0 compared to Fresh samples are shown, with significantly different taxa (Benjamini–Hochberg correction, FDR < 0.05) colored in red. The genera belonging to the core 95% group are indicated in bold, and the genera that have previously been associated with CRC are surrounded with a box. *FR* fresh-frozen samples; *FIT0* immediately frozen FIT samples; *SB0* immediately frozen stabilization buffer samples.

To test the differences in abundance among different genera, we used ALDEx2 to identify differentially abundant genera between fresh-frozen samples and FIT and stabilization buffer samples. The genera whose prevalence in all of the samples was less than 10% were filtered out, retaining 171 out of 360 genera for the analysis. Out of 171 genera analyzed, we observed 7 genera (4%) with significant differences in abundance between the FR and FIT0 samples and 16 genera (9.4%) with differences in abundance between the FR and SB0 samples (Fig. 4, Supplementary Table S5). The differences in abundance among the rest of the genera were not significant after correction for multiple testing (FDR > 0.05). Six genera with significant differences in abundance between the FIT0 and FR samples (*Alistipes*, *Anaerostipes*, *Eubacterium coprostalinogenes* group, *Romboutsia*, and uncultured *Ruminococcaceae)* also showed significant differences in abundance between SB0 samples and the FR samples, but only the *Eubacterium coprostanoligenes* group showed a change in the same direction for FIT0 and SB0.

Next, we identified 21 genera that belonged to the core microbiome (the genera present in over 95% of the samples) (Supplementary Table S5). Thereafter, we investigated which of all significantly different genera belonged to the core microbiome and were therefore common in our sample set. In the FIT samples, 6 out of 7 significantly different genera belonged to the core microbiome. In the stabilization buffer samples, 10 out of 16 genera belonged to the core microbiome. The significantly different genera belonging to the core group are indicated in bold in Fig. 4.

Although 16S sequencing does not provide species-level annotation, we compared the genera of the species previously known to be associated with CRC stages according to the multipopulation studies of Wirbel et al. 2019 and Yachida et al. 2019 and analyzed whether any of these genera differed significantly in our sample types (Supplementary Table S6)^11,12^*.* Among all of the taxa showing significant differences in the FIT0 samples relative to the fresh-frozen samples, none were found to be cancer related (Fig. 4). In the SB0 samples, cancer-related genera such as *Bacteroides* and *Dorea* presented significant differences relative to the fresh-frozen samples (Fig. 4). We did not detect *Fusobacteria,* which is often associated with CRC, among the genera that were present in 10% of the samples, indicating that this genus is not common among Estonian individuals who self-report being healthy.

#### Gut microbiome composition stability over time

**Figure 5:**
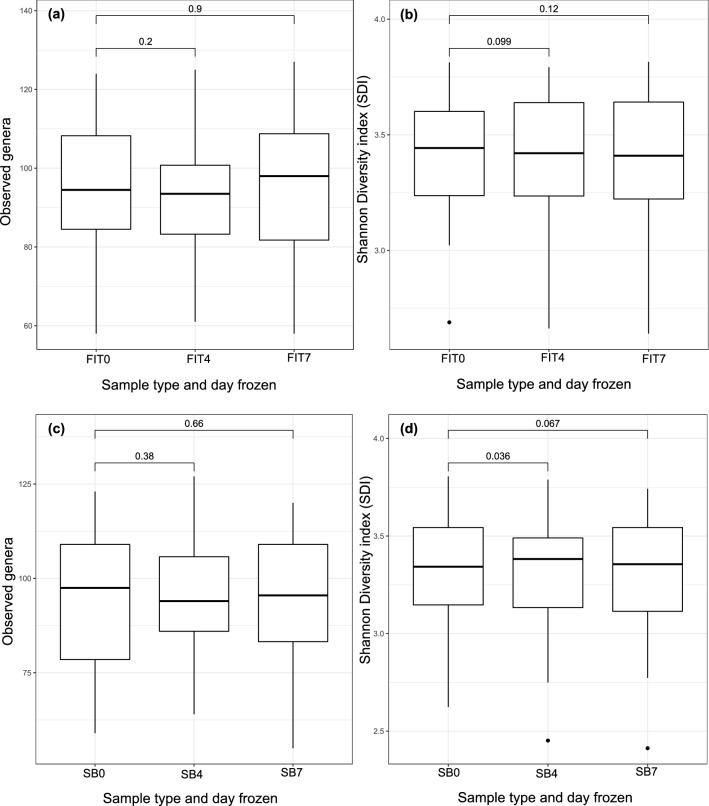
Microbiome diversity between different time points across sample types. Boxplots represent observed the genera (**a**) and Shannon diversity indexes (**b**) of FIT samples and the observed genera (**c**) and Shannon diversity indexes (**d**) of stabilization buffer samples from different time points. *FIT0* immediately frozen FIT samples; *FIT4* FIT samples frozen on day 4; *FIT7* FIT samples frozen on day 7; *SB0* immediately frozen stabilization buffer samples; *SB4* stabilization buffer samples frozen on day 4; *SB7* stabilization buffer samples frozen on day 7.

Next, we analyzed whether the microbiome composition remained stable in the FIT tubes and stabilization buffer after holding the samples at room temperature for 4 or 7 days (Fig. 1). For this purpose, FIT0 samples were compared to FIT4 and FIT7 samples, and SB0 samples were compared to SB4 and SB7 samples. The alpha diversity, beta diversity and differential abundance of the genera were analyzed. We detected no significant differences between the FIT samples frozen on different days in terms of the number of observed genera (FDR_FIT0-FIT4_ = 0.2; FDR_FIT0-FIT7_ = 0.9) (Fig. 5a) or Shannon diversity (FDR_FIT0-FIT4_ = 0.099; FDR_FIT0-FIT7_ = 0.12) (Fig. 5b) (Supplementary Table S4). *Romboutsia* was the only genus to show significant differences in abundance in the samples frozen on day 4 and day 7 relative to the immediately frozen FIT samples (FDR < 0.05) (Supplementary Table S7).

Similarly, no significant differences in the number of observed genera were detected between the SB0 and SB4 or SB7 samples (FDR_SB0-SB4_ = 0.38; FDR_SB0-SB7_ = 0.66) (Fig. 5c); however, the Shannon index was significantly lower in SB0 than in SB4 (FDR_SB0-SB4_ = 0.036; FDR_SB0-SB7_ = 0.067) (Fig. 5d) (Supplementary Table S4). No genera showed significant differences between the SB0 and SB4 or SB7 samples (Supplementary Table S7).

When we included all samples from all time points in the visualization of beta diversity, we observed that the samples remained clustered based on the individual subject, indicating that the interindividual differences were larger than those among different storage conditions and the days on which the samples were frozen (Supplementary Fig. S1). This was also supported by the PERMANOVA results, which showed that the day when the sample was frozen was not significant (R^2^ = 0.0012, *p* > 0.05). Furthermore, the results were insignificant when the interaction between sample type and frozen day was taken into account (R^2^ = 0.0011, *p* > 0.05).

## Discussion

CRC screening programs all over Europe use fecal immunochemical tests (FITs) as a first step to detect CRC. As CRC-associated microbiome signatures have been obtained from fresh frozen stool samples from patients with different stages of cancer^11,12^, using the microbiome to improve CRC screening has become a topic of high interest. In the current study, we aimed to test whether the samples stored in the FIT tubes used in national CRC screening programs are also suitable for microbiome analysis. The particular FIT tube (QuikRead iFOB sampling set) employed in this study has not been previously used for microbiome analysis in human stool samples. We compared the microbial communities detected from FIT tubes with those from fresh-frozen samples. We also used samples collected in stabilization buffer (DNA/RNA Shield) for the comparison of an additional collection method, as stabilization buffers are often used when the collection of fresh-frozen samples is not feasible. Additionally, we evaluated whether the genera differed significantly under either of the collection methods had been previously associated with CRC in multicohort population studies. Finally, the stability of the microbiome profile over 4 and 7 days was studied in both FIT tubes and stabilization buffer.

Our results indicate that the microbial communities obtained from fresh-frozen samples and FIT tubes are highly similar. The analysis of microbial alpha diversity demonstrated that the number of genera did not significantly differ between the storage methods. Small differences were identified in the Shannon index for the microbiome, proving the FIT samples to be less diverse than the fresh-frozen samples. However, beta diversity analysis clearly showed that the differences between subjects were greater than the differences between different storage methods. This is in accordance with previous studies that have found interindividual differences to be greater than intraindividual differences under different collection methods^15,18^. This was further confirmed by the results of analysis of variance, which also indicated that the storage conditions had a minimal effect (~ 2%) on the obtained gut microbiome composition. Donor-specific factors such as diet, age, medication use, stool consistency and host genetics are all the likely underlying causes of the substantial interindividual beta diversity differences.

When analyzing changes in genus abundances, we found that the FIT tube samples contained a similar community to the fresh-frozen samples, as only 4% of the genera were significantly different between the two collection methods. Even though most of the significantly different genera belonged to the groups found in the core microbiome (i.e., present in 95% of the samples), none of them had been previously associated with CRC in multicohort population studies^11,12^. We detected 16 CRC-associated bacterial genera; however, none of them differed in frequency between the FIT and fresh-frozen samples (Supplementary Table S5). This finding supports the possibility of using FIT tubes to study microbial biomarkers related to CRC. However, to provide convincing evidence of the suitability of currently employed FIT tubes for the detection of CRC-associated microbiome signatures, additional studies with CRC patient samples are needed.

Next, we wanted to determine if the stability of the microbial community could be affected by a longer shipping time, as the time from the collection of the initial fecal sample to its arrival at the study center for occult blood testing can be up to a week. When analyzing the effect of the storage time, the analysis of variance indicated that the effect of the day when the samples were frozen was not significant. Furthermore, when the microbial communities of the FIT samples with different storage times were compared to those of samples immediately frozen in FIT tubes, no differences in alpha diversity values were detected. Again, beta diversity analysis illustrated that the interindividual differences were greater than intraindividual differences when the data from day 4 and day 7 samples were compared to those of the samples that were frozen immediately. Differential abundance analysis revealed only one genus (*Romboutsia)* that differed significantly in the day 4 and day 7 samples relative to the immediately frozen FIT samples, indicating that the abundance of the vast majority of the genera was stable for at least one week. This is in accordance with previous studies showing that FIT tubes can preserve the gut microbiome at moderate to excellent levels^15,18,19^ and that the collection method and time stored at ambient temperature explain only small amounts of variability (< 10%)^15^. These previous studies have, however, all used FIT tubes from other manufacturers (OC-Sensor and OC-Auto® FIT).

Our study also included samples stored in stabilization buffer. Previous studies have indicated that the use of stabilization buffer is necessary when samples cannot be frozen immediately, as certain bacterial taxa start to bloom in untreated samples after spending several days at room temperature^15,20^. We found that compared to the FIT samples the microbiome community in the stabilization buffer samples was slightly less similar to the community in fresh-frozen samples. Although the community was relatively similar to that in fresh-frozen tubes in terms of microbial diversity and remained stable up to 7 days, the number of significant differences was higher for CRC-related bacteria, the core genera and the number of significantly different genera relative to the differences found between the FIT and fresh-frozen samples. Additionally, Shannon diversity was significantly lower compared to fresh-frozen samples as well as between SB0 and SB4 samples.

In summary, our results show for the first time that QuikRead iFOB sampling set tubes are suitable for storing fecal samples for microbiome studies, as the captured microbiome profile is similar to that obtained from fresh-frozen samples and remains stable for up to 7 days. However, the actual ability to detect cancer-specific bacterial signatures with this collection method needs to be confirmed in the future based on specific phenotypes, such as FIT positivity in patients with and without CRC, among other diseases that were included at this stage of our study. Additionally, the volunteers participating in our study self-reported their health status but did not undergo colonoscopy to confirm the healthy state of their gut. The analysis of the CRC-specific microbiome profile from the same FIT tubes used for fecal occult blood testing would allow to improve the detection of CRC with additional microbiome-based biomarkers. This could potentially make CRC diagnostics more sensitive and cost efficient. Improved screening not only improves the clinical outcome but also has significant socioeconomic benefits, as the healthcare costs related to CRC are considerable^21^. Fecal samples from CRC screening programs could be an excellent resource for biomarker discovery, which might lead to earlier detection of cancer or prior to its onset (in a precancerous state). The stool samples from FIT tubes could also be used in studying the role of the gut microbiome in other digestive diseases previously associated with the gut microbiome, such as pancreatic cancer^22^, inflammatory bowel disease and cholangitis^23^, as well as in population studies, in which samples are often sent via post and using fresh-frozen samples is not possible. Future studies should also investigate the possibility of using stool samples collected in FIT tubes for metagenomics and metabolomics analyses, which could provide additional information for early CRC detection.

## Materials and methods

### Sample population and collection

We enrolled 30 volunteers (16 women and 14 men) without known gastrointestinal disorders and who had not taken antibiotics within 60 days prior to sampling based on self-report questionnaires. All recruited subjects were Estonians aged between 22 and 68 (39 ± 12.1) years with BMIs ranging from 18.4 to 41.8 kg/m^2^ (mean 24 ± 4.7 kg/m^2^). Informed consent was obtained from the volunteers, and the study followed sampling protocols approved by the Ethics Committee of the University of Tartu.

Seven fecal samples were self-collected by each volunteer, and 214 samples (including positive and negative controls) were analyzed in this study. All the samples from the volunteers were collected within the same week (January 2020). A fresh stool sample was collected immediately after defecation with a sterile Pasteur pipette, placed inside a 15 ml conical polypropylene tube without any preservative and then frozen at − 20 °C (the gold standard for microbiome studies). From each fecal sample, 3 subsamples from each individual were transferred to QuikRead go iFOB fecal immunochemical test tubes (Aidian, Espoo, Finland) using a stick attached to the lid according to the instructions provided in the kit. Additionally, 3 subsamples were transferred to 1 ml tubes containing stabilization buffer (DNA/RNA Shield, Zymo Research, Irvine, California) using sterile swabs. To assess how storage time affects the stability of the community structure, one FIT tube and one stabilization buffer tube were instantly frozen together with the fresh stool sample. These tubes were frozen within 16 min (SD ± 16.9) after the sample was taken. Additional FIT and stabilization buffer tubes were stored at room temperature for either 96 h (4 days) or 168 h (7 days) and were then frozen at − 20 °C. The rationale for this step was that in CRC screening programs, the time from collecting the initial fecal sample until arriving at the study center for occult blood testing can be up to a week. Therefore, we wanted to see if longer shipping times could compromise the stability of the microbial community and affect the results of microbiome analysis. Furthermore, in addition to the ZymoBIOMICS™ Microbial Community Standard or MOCK (Zymo Research, Irvine, California), used as a positive control for sequencing, negative controls for each sample type were included in the DNA extraction and sequencing steps. The workflow for the sample collection and storage procedures is shown in Fig. 1.

### DNA extraction and sequencing

DNA extraction from all samples was performed using a Qiagen DNeasy PowerSoil Pro DNA extraction kit (Qiagen, Venlo, The Netherlands). For fresh-frozen samples, approximately 200 mg of stool was used as the starting material, following the DNA extraction kit manufacturer’s instructions, except that the samples were incubated for an additional 10 min at 65 °C after adding solution CD1 to ensure the proper lysis of difficult-to-lyse bacterial cells. The cell disruption step was performed using a Precellys 24 tissue homogenizer (parameters: 2 × 30 s, 2500 rpm, 30-s pause) (Bertin Instruments, Montigny-le-Bretonneux, France). For the samples stored in the stabilization buffer, 250 μL of liquid was used as the starting material. The rest of the protocol was the same as that for fresh-frozen samples. For the samples in the FIT tubes, up to 2 ml of the FIT solution was transferred to a new tube, and 200 μL of 1 M Tris–HCl, pH 7.5, was added to quench the formaldehyde present in the FIT solution. After centrifugation, the supernatant was discarded, and the pellet resuspended in CD1 solution was transferred to PowerSoil Pro tubes. To increase the DNA yield, decrosslinking was performed by 4 h of incubation at 65 °C with Proteinase K before the cell disruption step. The rest of the protocol was performed following the manufacturer’s instructions. DNA was quantified in all samples on a Qubit Fluorometer using a dsDNA HS Assay Kit (Thermo Fisher Scientific) and was then diluted to 5 ng/μL for sequencing. The DNA extraction protocol was also performed using negative controls (no solution as well as FIT and stabilization buffer tubes with the original solutions).

### Microbial community analysis

Amplicon sequencing was conducted as follows at the Institute of Genomics Core Facility, University of Tartu. The extracted DNA samples were quantified with a Qubit® 2.0 Fluorometer (Invitrogen, Grand Island, USA). Genomic DNA was amplified using the primers 16S_F (5′- TCGTCGGCAGCGTCAGATGTGTATAAGAGAC AGCCTACGGGNGGCWGCAG -3′) and 16S_R (5′-GTCTCGTGGGCTCGGAGATG TGTATAAGAGACAGGACTACHVGGGTATCTAATCC -3′) for the PCR amplification of an approximately 460 bp region within the hypervariable (V3-V4) region of the prokaryotic 16S ribosomal RNA gene^24^. Amplicon libraries for Illumina (Illumina, San Diego, USA) next-generation sequencing were generated by two-step PCR. First, a region specific for 16S rRNA was amplified via 24 cycles, and Illumina adapter and index sequences were then added via 7 cycles of PCR. The quality control of the amplicon libraries was performed via Agilent 2200 TapeStation analysis (Agilent Technologies, Santa Clara, USA) and with a Kapa Library Quantification Kit (Kapa Biosystems, Woburn, USA). The amplicon libraries were pooled in equimolar concentrations. Sequencing was carried out on an Illumina MiSeq System using a MiSeq Reagent Kit v3 in paired end 2 × 300 bp mode.

Raw sequences were demultiplexed with Illumina bcl2fastq2 Conversion Software v2.20. Bioinformatics analyses were performed using the open-source software QIIME2 (version 2019.7)^25^. Raw data were imported using the q2-tools import script with the PairedEndFastqManifestPhred33 input format. In total, 7 468 645 reads were generated (34 738 reads per sample on average). The total number of reads was 1 116 409 (37 214 reads on average) for fresh samples (FR); 1 048 723 (34 957 reads on average) for FIT Day 0 samples (FIT0); 1 066 370 (35 546 reads on average) for FIT Day 4 samples (FIT4); and 1 066 370 (34 222 reads on average) for FIT Day 7 samples (FIT7). For the DNA/RNA Shield stabilization buffer samples, the number of reads was 1 028 579 (34 286 reads on average) on Day 0 (SB0), 1 127 694 (37 590 reads on average) on Day 4 (SB4), and 1 026 705 (34 224 reads) on Day 7 (SB7).

 Denoising was performed using DADA2 software, which uses a quality-aware model of Illumina amplicon errors to obtain the abundance distribution of sequence variance, with a single-nucleotide difference^26^. Based on the obtained quality scores, the q2-dada2-denoise script was used to truncate the forward reads at position 250 and trim them at position 15, and reverse reads were truncated at position 247 and trimmed at position 12. Chimeras were removed using the “consensus” filter, which detects the chimeras in each sample individually. With this method, the sequences that were established as chimeric in a fraction of samples were removed. During the denoising steps, forward and reverse reads were also merged. Subsequently, the alignment of amplicon sequence variants (ASVs) was performed using MAFFT^27^. Thereafter, FASTTREE was used to construct the phylogenetic tree^28^. Taxonomy was assigned using the q2-feature classifier with the pretrained naïve Bayes classifier, which was based on the reference reads from the SILVA 16S V3-V4 v132_99 databases with a similarity threshold of 99%^29,30^. All samples passed the quality control (QC) procedure, and the negative controls, as expected, showed 0 ASVs after the quality control steps.

### Statistical analysis

Statistical analyses were carried out in RStudio (version 1.2.1335, R version 3.6.1) using the packages *phyloseq* (v.1.30.0)^31^, *microbiome* (v.1.8.0)^32^, *vegan* (v.2.5-6)^33^, stats (v.3.2.1) and *ALDEx2* (v.1.18.0)^34^. All visualizations were conducted using *ggplot2* (v.3.2.1)^35^. Alpha diversity metrics such as the number of genera (estimate of the richness of the sample) and the Shannon diversity index (takes into account both sample richness and evenness) were calculated based on the genus-level microbiome profile using the *phyloseq* package. Between-sample distances were calculated using the Euclidean distance metric based on a centered log ratio (CLR)-transformed genus-level microbiome profile^36^. Permutational analysis of variance (PERMANOVA) of between-sample distances was carried out to test whether differences in the microbial composition (beta diversity) were associated with the sample type and the time the sample spent at room temperature. PERMANOVA was performed using the *adonis* function of the *vegan* package (v.2.5-6.). The *Microbiome* package (v.1.6.0) was used to determine the core genera of the microbiome with a detection threshold of 0 and a prevalence threshold of 95%. Welch’s paired t test integrated in the ANOVA-Like Differential Expression tool (ALDEx2, v.1.18.0) was used for the differential abundance analysis of genera to assess whether the FIT or stabilization buffer samples differed from fresh-frozen samples (accuracy of different collection strategies) as well as whether there were differences between the samples frozen immediately and the samples frozen on day 4 or day 7 (stability over time). To limit the number of tests, the genera whose prevalence was less than 10% were filtered out, retaining 171 out of 360 genera for the analysis. Multiple testing was taken into account using the Benjamini–Hochberg false discovery rate (FDR) method, and *p*-values < 0.05 were considered to be statistically significant^37^.

## Supplementary Information

Supplementary Information 1.

Supplementary Information 2.

## Data Availability

The datasets generated during and/or analysed during the current study are available from the corresponding author on reasonable request.
