# Supplementary material for: Using fecal immunochemical tubes for the analysis of the gut microbiome has the potential to improve colorectal cancer screening
Source: Sci Rep. 2021 Oct 1;11:19603. doi: 10.1038/s41598-021-99046-w (PMC8486803; doi:10.1038/s41598-021-99046-w)
Supplement: Supplementary file 1 — Supplementary Information 1. [file 41598_2021_99046_MOESM1_ESM.pdf]

# Supplementary Information

## Using fecal immunochemical tubes for the analysis of the gut microbiome has the potential to improve colorectal cancer screening

Kertu Liis Krigul[1,2], Oliver Aasmets[1,2], Kreete Lüll[1,2], Tõnis Org[1,2], Elin Org[1*]

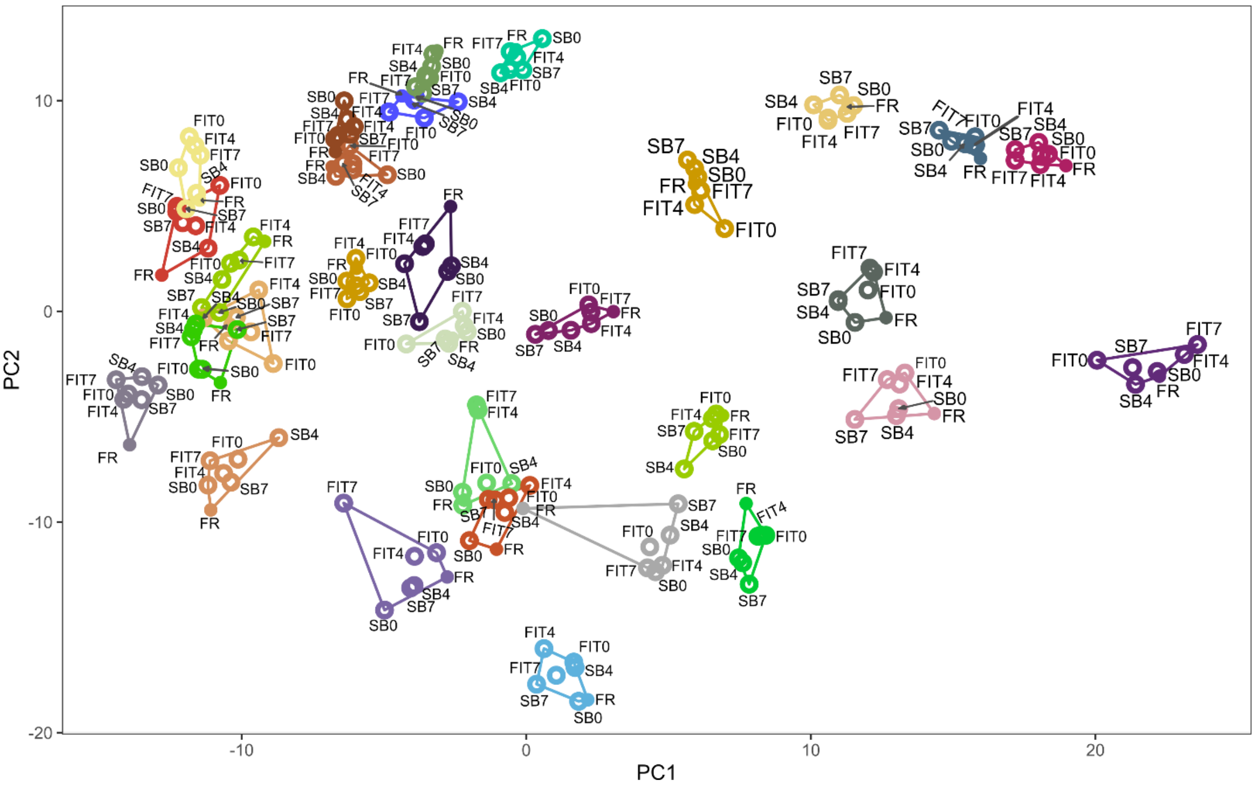


**Supplementary Figure S1. PCA plot showing the differences in the samples collected using different storage conditions including all samples taken from the volunteers.** Samples are colored and linked based on the Patient ID. Abbreviations: FR – fresh-frozen samples; FIT0 – immediately frozen FIT samples; FIT4 – FIT samples frozen on day 4; FIT7 – FIT samples frozen on day 7; SB0 – immediately frozen stabilization buffer samples; SB4 – stabilization buffer samples frozen on day 4; SB7 – stabilization buffer samples frozen on day 7.
